# Relationship between hippocampal subfield volumes and cognitive decline in healthy subjects

**DOI:** 10.3389/fnagi.2023.1284619

**Published:** 2023-12-07

**Authors:** Simon Doran, Daniel Carey, Silvin Knight, James F. Meaney, Rose Anne Kenny, Céline De Looze

**Affiliations:** ^1^Department of Radiology, St James’s Hospital, Dublin, Ireland; ^2^The Thomas Mitchell Centre for Advanced Medical Imaging, St James’s Hospital, Dublin, Ireland; ^3^The Irish Longitudinal Study on Ageing, School of Medicine, Trinity College Dublin, Dublin, Ireland; ^4^The Mercer’s Institute for Successful Ageing (MISA), St James’s Hospital, Dublin, Ireland

**Keywords:** hippocampus subfields, MRI, cognitive decline, risk factors, dementia

## Abstract

We examined the relationship between hippocampal subfield volumes and cognitive decline over a 4-year period in a healthy older adult population with the goal of identifying subjects at risk of progressive cognitive impairment which could potentially guide therapeutic interventions and monitoring. 482 subjects (68.1 years +/− 7.4; 52.9% female) from the Irish Longitudinal Study on Ageing underwent magnetic resonance brain imaging and a series of cognitive tests. Using *K*-means longitudinal clustering, subjects were first grouped into three separate global and domain-specific cognitive function trajectories; High-Stable, Mid-Stable and Low-Declining. Linear mixed effects models were then used to establish associations between hippocampal subfield volumes and cognitive groups. Decline in multiple hippocampal subfields was associated with global cognitive decline, specifically the presubiculum (estimate −0.20; 95% confidence interval (CI) -0.78 – −0.02; *p* = 0.03), subiculum (−0.44; −0.82 – −0.06; *p* = 0.02), CA1 (−0.34; −0.78 – −0.02; *p* = 0.04), CA4 (−0.55; −0.93 – −0.17; *p* = 0.005), molecular layer (−0.49; −0.87 – −0.11; *p* = 0.01), dentate gyrus (−0.57; −0.94 – −0.19; *p* = 0.003), hippocampal tail (−0.53; −0.91 – −0.15; *p* = 0.006) and HATA (−0.41; −0.79 – −0.03; *p* = 0.04), with smaller volumes for the Low-Declining cognition group compared to the High-Stable cognition group. In contrast to global cognitive decline, when specifically assessing the memory domain, cornu ammonis 1 subfield was not found to be associated with low declining cognition (−0.14; −0.37 – 0.10; *p* = 0.26). Previously published data shows that atrophy of specific hippocampal subfields is associated with cognitive decline but our study confirms the same effect in subjects asymptomatic at time of enrolment. This strengthens the predictive value of hippocampal subfield atrophy in risk of cognitive decline and may provide a biomarker for monitoring treatment efficacy.

## Introduction

With an increase in the ageing world population, prevention, diagnosis, and treatment of cognitive impairment is of significant public interest. Hippocampal degeneration is strongly associated with Alzheimer’s type dementia (AD), the most prevalent form of dementia, affecting 60–80% of cases (Braak and Braak, 1991; Hebert et al., 2003; Apostolova et al., 2006; Scott and Barrett, 2007; Pluta et al., 2012; La Joie et al., 2013; Kälin et al., 2017). Mild Cognitive Impairment (MCI), a potential precursor of Alzheimer’s dementia, has a similar histopathological basis (Kälin et al., 2017). The conversion rate from MCI to AD is in approximately 10–15% per year with a 6- year conversion rate of up to 80% (Petersen et al., 1999, 2009). Identifying subjects at risk of progressive cognitive impairment could allow future targeted therapeutic interventions and closer monitoring of this cohort.

The link between hippocampal atrophy and cognitive impairment is well established. Reduced whole hippocampal volume (WHV) has been associated with global cognitive impairment (Fan et al., 2007; Tondelli et al., 2012) and more specifically verbal memory impairment in cross-sectional (Desikan et al., 2009) and longitudinal studies (De Leon et al., 2006; Devanand et al., 2007; Costafreda et al., 2011; Westman et al., 2011; Izzo et al., 2020), typically in patients with diagnosed cognitive impairment and/or dementia. Reduced WHV has also been associated with preclinical MCI/AD and clinical MCI/AD in cross-sectional (Li et al., 2007; Desikan et al., 2009) and longitudinal studies (De Leon et al., 2006; Devanand et al., 2007; Fan et al., 2007; Costafreda et al., 2011; Westman et al., 2011; Tondelli et al., 2012). In addition, WHV has been used for the classification of cognitively normal vs. AD and for predicting progressive MCI with accuracy rates ranging from 85 to 94.3% (Fan et al., 2007; Costafreda et al., 2011) and 79 to 87% (Devanand et al., 2007; Fan et al., 2007; Westman et al., 2011) respectively.

Within the hippocampus, subfields are variably affected by pathological processes and thus the entire hippocampal volume may mask subfield volume changes. For example, cornu ammonis 1 (Ca1) is prone to the accumulation of pathological neurofibrillary tangles (Braak and Braak, 1991) whilst the parasubiculum is typically not involved in the degenerative process (Zheng et al., 2018).

Several studies have investigated hippocampal subfield volumes in MCI and AD (Csernansky et al., 2005; Apostolova et al., 2006, 2010; Hanseeuw et al., 2011; Pluta et al., 2012; La Joie et al., 2013; Khan et al., 2015; Izzo et al., 2020; Kwak et al., 2022). Reduced CA1, subiculum and presubiculum subfield volumes have been found in MCI/AD subjects compared to healthy controls in cross-sectional studies (Pluta et al., 2012; La Joie et al., 2013) and longitudinal studies (Csernansky et al., 2005; Apostolova et al., 2006; Khan et al., 2015). The CA1, presubiculum, subiculum and fissure have been shown to be sensitive markers of conversion from MCI to AD (Khan et al., 2015; Izzo et al., 2020) and for identifying individuals with MCI compared to WHV (Pluta et al., 2012; La Joie et al., 2013; Khan et al., 2015). Most studies examine subjects with diagnosed MCI or AD and compare them to healthy controls, but studies using a healthy cohort of older adults as presented in this paper are lacking. Using a large subsample of a nationally representative cohort of older adults, The Irish Longitudinal Study on Ageing (TILDA), Carey et al. (2019) observed cross-sectional associations between verbal episodic memory (immediate and delayed word recall) and the hippocampal subfield volume of CA1, CA2/3, CA4, molecular layer and granule cell layer of dentate gyrus (GCMLDG). In this study, using TILDA data, we build on Carey’s work and examine the relationship between hippocampal subfield volumes and cognitive decline over a 4-year period in initially normal subjects. We hypothesised that lower hippocampal subfield volumes, in particular the CA1, subiculum, CA3, CA4 and GCMLDG subfields, are associated with a faster rate of cognitive decline, specifically in the memory domain.

## Methods

### Ethics statement

This study was approved by the Trinity College Faculty of Health Sciences Research Ethics Committee, Dublin, Ireland. Protocols conformed with the Declaration of Helsinki. Signed informed consent was obtained from all respondents prior to participation. Additional ethics approval was received for the magnetic resonance imaging (MRI) sub-study from the St. James’s Hospital/Adelaide and Meath Hospital, Inc. National Children’s Hospital, Tallaght (SJH/AMNCH) Research Ethic Committee, Dublin, Ireland. Those attending the MRI sub-study also completed an additional MRI-specific consent form.

### Study design

The Irish Longitudinal Study on Ageing (TILDA) is a prospective nationally representative study of older adults living in the community in the Republic of Ireland (ROI). Its design has been previously outlined (Whelan and Savva, 2013). Briefly, participants were recruited from a clustered stratified random sample of the population from across the Republic of Ireland, aged 50 or over at the time of recruitment. Multiple waves of data collection have been undertaken during the lifetime of TILDA. In this paper we analysed data collected as part of wave 3 (2014–2015), 4 (2016) and 5 (2018–2019). At each wave, participants took part in a computer assisted personal interview (CAPI) and completed a questionnaire (SCQ). At Wave 3, participants were also invited to take part in a health assessment and a sub sample of those who completed the health assessment were randomly selected to undergo magnetic resonance imaging (MRI).

### MRI acquisition

The MRI sampling procedure has been previously described in detail (De Looze et al., 2020). Briefly, images were acquired using a 3-Tesla Philips Achieva system and a 32-channel head coil at the Thomas Mitchell Centre for Advanced Medical Imaging (CAMI) based in St James Hospital, Dublin. For volumetric analysis, high resolution T1 weighted (T1W) images were acquired. The specific parameters of the acquired images are: FOV (mm): 240 × 218 × 162; 0.9 mm isotropic resolution; SENSE factor: 2; TR: 6.7 ms; TE: 3.1 ms; flip angle: 9°. 578 participants were enrolled for MRI imaging. Initial recruitment focused on individuals aged 65 and over with later recruitment targeting the 50–64 years age group. Images were acquired on 560 individuals (18 subjects did not proceed with the scan for anxiety/claustrophobia (*n* = 14) and contraindications (*N* = 4) to MRI). 58 scans were excluded due to motion artefacts (*N* = 33), grey matter/white matter lesions (*N* = 18) and participants reporting a medical history of transient ischemic attack or stroke (*N* = 7). 482 participants had MRI data and cognitive tests available at Wave 3 and Wave 4 or 5 for analysis. Supplementary Figure S1 presents the flowchart of the study sample.

### MRI analysis

All T1w images were analysed using FreeSurfer (version 6.0), a validated, automated image analysis software (Fischl et al., 2002; Fischl, 2012; Iglesias et al., 2015; Zandifar et al., 2017). The hippocampal subfield option was used to segment the hippocampus into its subfields (Yushkevich et al., 2015a,b; Carey et al., 2019). Briefly, the procedure uses probabilistic modelling based on *ex-vivo* hippocampus analysis and T1W *in-vivo* image analysis to divide the hippocampus into 12 subfields: the presubiculum, parasubiculum, subiculum, cornu ammonis 1 (CA1), cornu ammonis 3 (CA3), cornu ammonis 4 (CA4), molecular layer, hippocampal amygdala transition area (HATA), granule cell layer of the dentate gyrus (GCMLDG), fimbria, tail, and fissure. Segmentations were validated by trained operators. All data fell within expected boundaries. Volumetric data (mm^3^) was generated for each hippocampal subfield and left and right sides were added for analysis. Estimated total intracranial volume (eTIV) was calculated using the FreeSurfer “recon-all” processing function.

### Cognitive function

Global cognitive function collected during the personal interviews at each wave was assessed using the Mini Mental State Exam (MMSE), a 30-point test validated for dementia screening. Verbal memory was assessed using a 10-word delayed recall task and verbal fluency/executive function was measured using animal naming. Details on the individual tests are published elsewhere (Cronin et al., 2013). Trajectories of global cognitive function and domain-specific function were estimated using *K*-means cluster modelling for longitudinal data (kml package in R version 4.0.3 with RStudio Version 1.4.1103). The kml analysis was specified to allow between 2 and 5 clusters (group-based trajectories), each obtained by running 1,000 permutations. The optimum number of clusters (groups) was determined using the Calinski and Harabasz criteria (Genolini et al., 2015), along with consideration of clinical relevance. Three groups of global cognitive function were identified: the high-increasing (65%), the mid-stable (29%), and the low-declining (6%). Supplementary Figure S2 provides the MMSE means and standard deviations (SDs) per wave and cognitive function trajectory group. Supplementary Figure S3 illustrates the cognitive function trajectories per group from Wave 3 to Wave 5. The mean and standard deviations (SDs) per wave and per cognitive domain trajectory group are given in Supplemental material (see Supplementary Tables S4–S6).

### Covariates

Covariates included age, sex, education (none/primary, secondary, tertiary/higher). Pre-existing self-reported cardiovascular diseases and events (CVDs) included a history of angina, myocardial infarction, heart failure, atrial fibrillation, transient ischaemic attack, and ischaemic cerebrovascular attack (stroke). The CVDs variable was a pooled binary measure with an outcome of either absent (CVDs = 0) or present (CVDs > = 1). Seated systolic (SBP) and diastolic (DBP) blood pressure measurements were obtained separated by 1 min using an OMRON™ digital automatic blood pressure monitor (Model M10- IT). Smoking status was categorized as never, former, or current. Alcohol misuse was measured using the CAGE questionnaire and represented as a binary variable (Mayfield et al., 1974). Physical activity was assessed through the International Physical Activity Questionnaire (IPAQ) (Hagströmer et al., 2006) as a three-level variable (high/moderate/low). Body mass index (BMI) (kg/m^2^) was calculated; height and weight were measured to the nearest 0.01 m and 0.1 kg, respectively. The 8-item Centre for Epidemiologic Studies Depression Scale (CESD-8, short form) (Radloff, 1977) was used to measure depressive symptoms, with scores ranging from 0–24. Medications were categorized as per the anatomical therapeutic chemical (ATC) classification codes. Binary antihypertensive (ATC C02, C03, C07, C08 and C09) and antidepressant medication (ATC N06A) was used in the analysis.

### Statistical analysis

#### Data descriptives

The observed sample was first characterized by baseline demographics, diseases, lifestyle factors and medications. Continuous variables were described as unadjusted means with SDs; categorical variables were given as percentages (%). To estimate selection bias, the observed sample was also compared to the excluded cohort. *t*-tests and chi-squared tests (where appropriate) were used to assess differences between the two groups.

#### Hippocampal subfields and global cognitive decline

Multilevel mixed effects linear modelling was used to assess the relationship between hippocampal subfields and four-year global cognitive decline (MMSE). Hippocampal subfield volumes were the dependent variable. Fixed effects were the global cognitive function trajectory groups (High-Stable set as the reference level) and hippocampal subfields (the parasubiculum set as the reference level), with an interaction term. The *significance* of the *interaction term was assessed* through *likelihood ratio tests*. Participants constituted the random intercept (models had a nested structure such that the different hippocampal subfields were nested within each participant). Baseline models included age, sex, education, and head size as covariates. Full models were further adjusted for self-reported CV diseases, blood pressure, lifestyle factors (smoking, alcohol consumption, physical activity, bmi), depressive symptoms and medications (antihypertensives and antidepressants).

#### Supplementary analyses

We also examined the relationship between hippocampal subfields and domain specific (verbal memory and verbal fluency) cognitive decline. The same analyses as described above were repeated with trajectory groups of immediate recall, delayed recall and verbal fluency used as fixed effects in separate models.

## Results

### Characteristics of the observed sample

Table 1 provides descriptive statistics of the study sample in comparison with the rest of the cohort (excluded participants) at Wave 3. Mean age was 68.1 years (SD = 7.4 years) and 52.9% of participants were female. Compared to the excluded cohort, participants in the study sample were older (*p* < 0.001) with a higher education level (p < 0.001). They had lower diastolic blood pressure (*p* = 0.03) and BMI (*p* = 0.009) and were less likely to be presently smoking (*p* < 0.001). They also had lower levels of depressive symptoms (*p* = 0.001) and higher global (*p* = 0.001) and domain-specific (*p* < 0.01) cognitive scores compared to the excluded cohort (delayed recall *p* = 0.001 and verbal fluency *p* = 0.01).

**Table 1:** Characteristics of the study sample with reference to the excluded TILDA cohort.

	Study sample (*N* = 482)	Excluded (*N* = 6,202)
Age (mean, sd)	68.1 (7.4)^***^	66.5 (9.7)
Sex (Female, %)	52.9	56.2
Education (Low, %)	19.5^***^	26.5
CVD conditions (>1, %)	61.4	62.6
Systolic/ Diastolic BP (mean, sd)	133.7 (18.5) / 79.7 (10.7)^*^	134.1 (19.5)/ 80.8 (10.3)
Antihypertensives (on meds, %)	40.2	43.7
BMI (mean, sd)	27.9 (4.6)^**^	28.5 (5.1)
Smoking (Present, %)	6.4^***^	13.2
Physical activity (low, %)	33.6	40.4
Problematic alcohol (%)	8.9	12.5
Depressive symptoms (mean, sd)	3.6 (3.5)^**^	4.2 (3.9)
Antidepressants (%)	6.4^*^	9.6
MMSE (mean, sd)	28.8 (1.4)^**^	28.4 (2.1)
Delayed Recall (mean, sd)	6.4 (2.4)^**^	5.9 (2.6)
Verbal Fluency (mean, sd)	19.5 (5.4)^*^	18.8 (6.0)

*t*-tests and chi-squared tests (where appropriate) were used to assess differences between the two groups. ^***^p < 0.001, ^**^p < 0.01, ^*^p < 0.05.

### Hippocampal subfields and global cognitive decline

The interaction between cognitive groups and multiple hippocampal subfields in the baseline model was significant (χ^2^_(42)_ = 51.0, *p* < 0.001). Significant cognitive decline by subfield interactions emerged for the presubiculum (*p* = 0.03), subiculum (*p* = 0.02), CA1 (*p* = 0.04), CA4 (*p* = 0.005), molecular layer (*p* = 0.01), dentate gyrus (*p* = 0.003), hippocampal tail (*p* = 0.006) and HATA (p = 0.04) with smaller volumes for the Low-Declining group compared to the High-Stable group. The Mid-Stable group also had smaller presubiculum (*p* = 0.04), hippocampal tail (*p* = 0.04) and HATA (*p* = 0.02) volumes compared to the High-Stable group.

**Figure 1:**
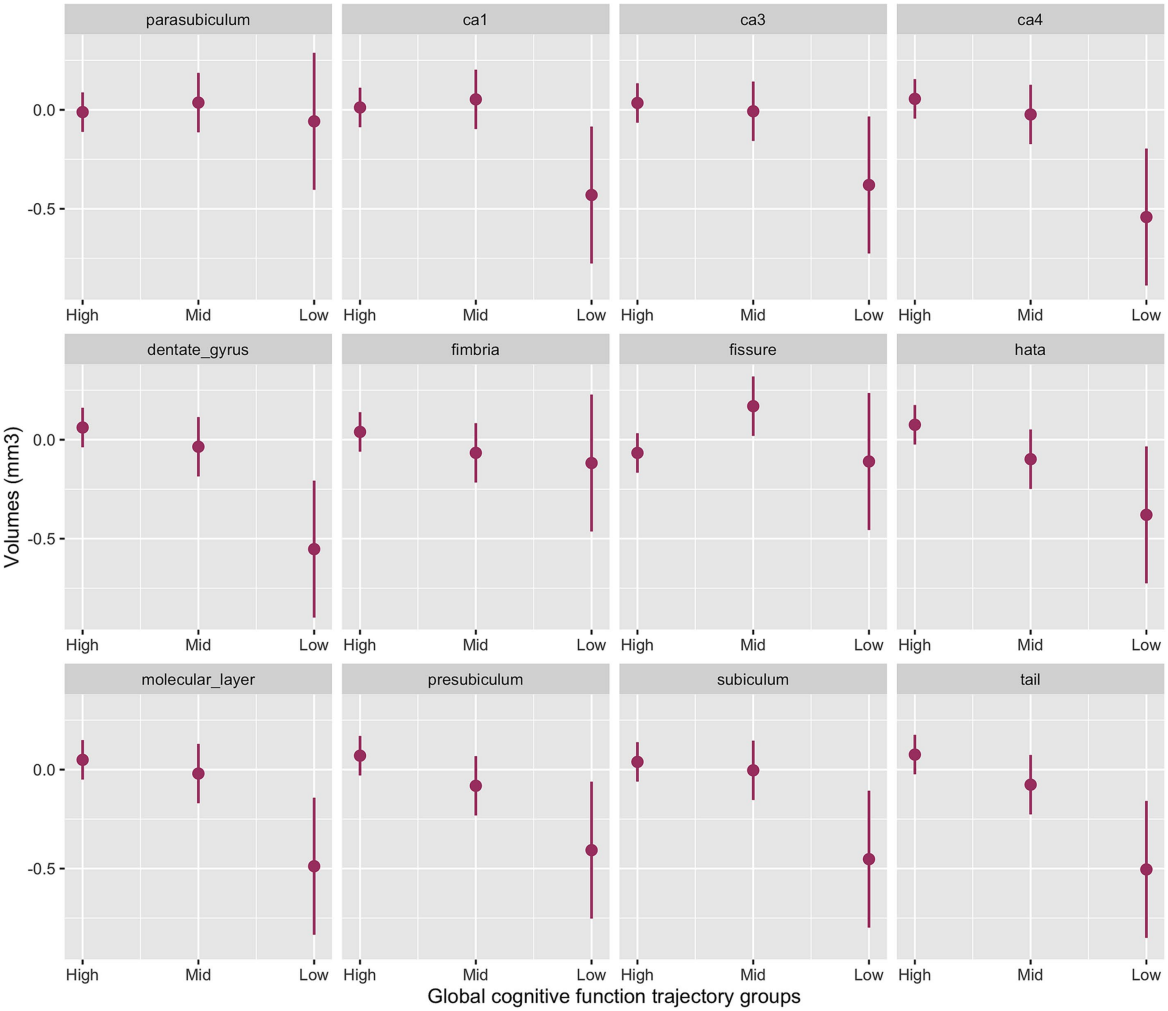
Baseline model output of marginal estimates of hippocampal subfield volumes across the three global cognitive function trajectory groups. High, High-Stable group; Mid, Mid-Stable group; Low, Low-Stable group; Ca1, cornu ammonis 1; Ca3, cornu ammonis 3; Ca4, cornu ammonis 4; hata, hippocampal amygdala transition area; tail, hippocampal tail.

Figure 1 provides the marginal estimates (95% CI) of hippocampal subfield volumes across High-Stable, Mid-Stable, and Low-Declining cognitive function trajectory groups adjusted for age, sex, education and eTIV. See Supplementary Table S4 for the baseline model output. These associations remained significant in the fully adjusted model (Supplementary Table S5).

### Supplementary analyses

#### Hippocampal subfields and verbal memory

The interaction between memory cognitive groups and multiple hippocampal subfields in the baseline model was significant (χ^2^_(42)_ = 63.2, *p* < 0.001). Significant cognitive decline by subfield interactions emerged for the presubiculum (*p* = 0.003), subiculum (*p* = 0.02), CA3 (*p* = 0.04) CA4 (*p* = 0.008), molecular layer (*p* = 0.008), dentate gyrus (*p* = 0.004), hippocampal tail (*p* = 0.003), HATA (*p* = 0.001) and fimbria (*p* < 0.001) with smaller volumes for the Low-Declining group compared to the High-Stable group. The Mid-Stable group had larger fissure volume (*p* = 0.001) compared to the High-Stable group.

**Figure 2:**
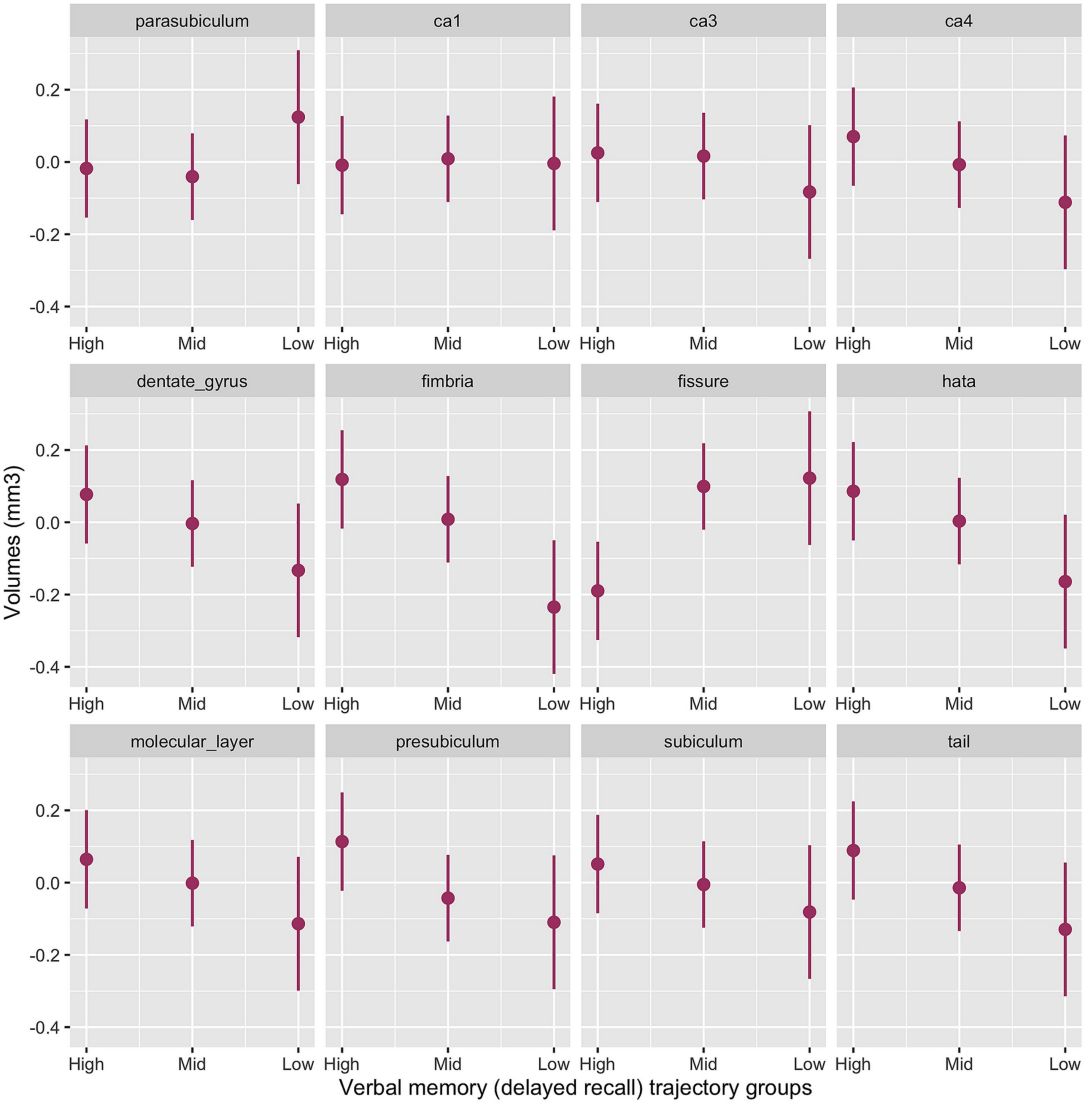
Baseline model output of marginal estimates of hippocampal subfield volumes across the three verbal memory cognitive trajectory groups. High, High-Stable group; Mid, Mid-Stable group; Low, Low-Stable group; ca1, cornu ammonis 1; ca3, cornu ammonis 3; ca4, cornu ammonis 4; hata, hippocampal amygdala transition area; tail, hippocampal tail.

Figure 2 provides the marginal estimates (95% CI) of hippocampal subfield volumes across High-Stable, Mid-Stable, and Low-Declining cognitive function trajectory groups adjusted for age, sex, education and eTIV. See Supplementary Table S6 for the baseline model output. These associations remained significant in the fully adjusted model (Supplementary Table S7).

#### Hippocampal subfields and verbal fluency

The interaction between cognitive groups and multiple hippocampal subfields in the baseline model was significant (χ^2^_(42)_ = 44.4, *p* = 0.003). Significant cognitive decline by subfield interactions emerged for the presubiculum (p = 0.02), CA1 (*p* = 0.05), CA3 (*p* = 0.02) CA4 (*p* = 0.007), molecular layer (*p* = 0.02), dentate gyrus (*p* = 0.006), hippocampal tail (*p* = 0.02) and HATA (*p* = 0.01) with smaller volumes for the Low-Declining group compared to the High-Stable group. The Mid-Stable group also had smaller CA3 volume (*p* = 0.04) compared to the High-Stable group.

**Figure 3:**
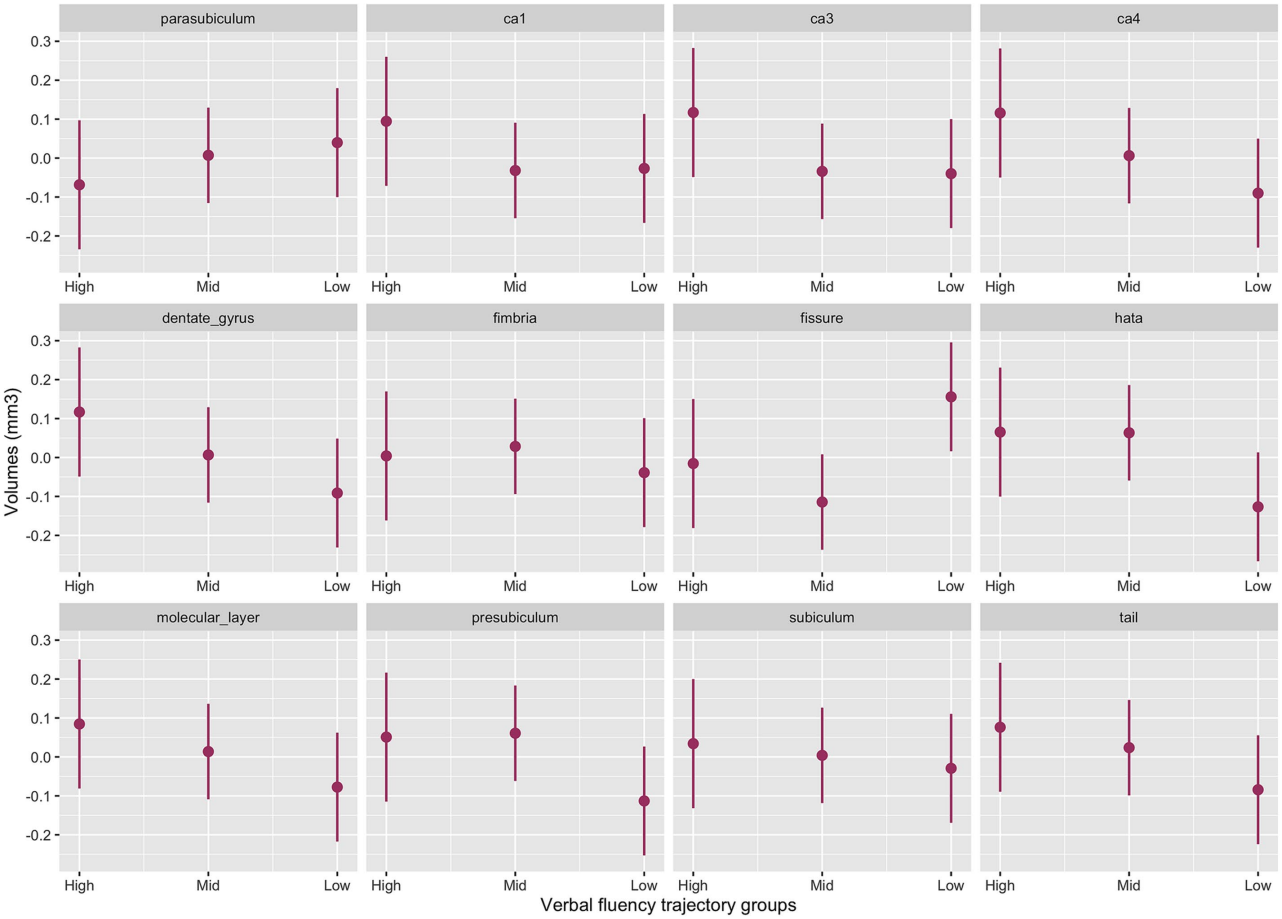
Baseline model output of marginal estimates of hippocampal subfield volumes across the three verbal fluency cognitive trajectory groups. High, High-Stable group; Mid, Mid-Stable group; Low, Low-Stable group; ca1, cornu ammonis 1; ca3, cornu ammonis 3; ca4, cornu ammonis 4; tail, hippocampal tail.

Figure 3 provides the marginal estimates (95% CI) of hippocampal subfield volumes across High-Stable, Mid-Stable, and Low-Declining cognitive function trajectory groups adjusted for age, sex, education and eTIV. See Supplementary Table S8 for the baseline model output. These associations remained significant in the fully adjusted model (Supplementary Table S9).

## Discussion

The hippocampus is a critical structure in memory retrieval and whole hippocampal atrophy has been well documented as a biomarker of cognitive impairment (De Leon et al., 2006; Devanand et al., 2007; Fan et al., 2007; Costafreda et al., 2011; Westman et al., 2011; Tondelli et al., 2012). Previous pathologic studies have shown that subfields are affected differentially in amnestic cognitive impairment due to neurofibrillary pathology preferentially affecting CA1 with a more delayed and less pronounced effect on CA2-3 (Braak and Braak, 1991). Prior studies have examined relationships between hippocampal subfield volumes and cognitive decline in cohorts with diagnosed MCI and/or dementia (Apostolova et al., 2006; Khan et al., 2015; Kälin et al., 2017; Izzo et al., 2020). In this study, we examined the relationship between hippocampal subfield volumes and global and domain specific cognitive decline over a four-year period in a relatively large MRI cohort of older subjects from a nationally representative population-based study, the Irish Longitudinal Study on Ageing. We hypothesised that due to its critical role in memory retrieval, subjects with declining cognition would have lower subfield volumes, particularly those subfields implicated in early cognitive impairment (CA1 and subiculum). We found that individuals with cognitive decline at follow-up have a widespread pattern of subfield atrophy including the presubiculum, subiculum, CA1, CA4, molecular layer, dentate gyrus, hippocampal tail and HATA subfields, all of which demonstrated lower volumes at baseline compared to those who remained stable over 4 years.

Our results broadly corroborate with published literature reporting total hippocampal volumes, but clarifies several important anatomical subfields that correlate with specific metrics of brain function. In a longitudinal study Khan et al. (2015) reported widespread subfield atrophy involving all segmented subfields in their study (CA1, CA2-3, CA4-DG, fimbria, presubiculum, subiculum). In another longitudinal study, Kälin et al. (2017) examining subfield volume differences between healthy controls and subjects with MCI who converted to AD (MCI-CB) and subjects who maintained their cognition level (MCI-S) found the presence of multi-subfield atrophy (CA1, CA4/DG, subiculum, strata; not CA2-3) in subjects with MCI-CB while subjects with MCI-S had similar subfield volumes to healthy controls. There are some limitations in comparison of published literature and the present study due to use of different hippocampal segmentation techniques, different cognitive measures across all studies and our focus on healthy older adults rather than subjects with a formal MCI or AD diagnosis as was examined in all previous studies. Notwithstanding these limitations, the same trend for reduced subfield volumes is a consistent finding both in cognitively normal subjects who develop cognitive impairment and those with early MCI.

In our supplementary analyses of verbal memory and verbal fluency in patients with cognitive dysfunction, we identified a multi-subfield pattern of volume loss.

Firstly for verbal episodic memory, subfield atrophy involved the presubiculum, subiculum, CA3, CA4, molecular layer, dentate gyrus, hippocampal tail, HATA and fimbria. This pattern was more widespread than identified by some previous studies including Zammit et al. (2017) in a cross-sectional study using a verbal memory test (Free and Cued Selective Reminding Test Delayed Free Recall) (which found lower volumes only in the CA1 and subiculum subfields), In a much smaller (*n* = 47) cross-sectional study of healthy volunteers (similar to our patient population although with a higher proportion of young adults) Aslaksen et al. (2018) found volume loss in CA1, CA2/3 and CA4/DG to be associated with reduced verbal episodic memory scores. Our results strengthen the findings of a recent cross-sectional study published by our research group, examining the relationship between recall memory and hippocampal subfield volumes, finding that the GCMLDG and the molecular layer are associated with impaired verbal episodic memory (Carey et al., 2019) although again we identified a more widespread pattern of volume loss.

Discordant to the published literature, and in contrast to our hypothesis, we found that the CA1 subfield was not associated with verbal episodic memory decline. This is despite cumulative evidence supporting CA1 to be a critical structure in memory retrieval decline (Apostolova et al., 2006, 2010; Kerchner et al., 2012; Carlesimo et al., 2015; Khan et al., 2015; Kälin et al., 2017; Zammit et al., 2017). Our finding is consistent with a recent study by Izzo et al. (2020), who reported no association between verbal memory and cognitive decline. Verbal episodic memory impairment is the usual subjective memory complaint that portends a diagnosis of MCI (Gifford et al., 2015). Therefore, as previous studies have generally tended to use a clinical diagnosis of MCI, along with global cognitive assessments such as the MMSE and Montreal Cognitive Assessment (MOCA) to subdivide participants and evaluate cognition, it would be reasonably expected that CA1 would be associated with a declining DR score. One reason for discordance may be the difficulty in segmenting out the subiculum and CA1 as they are anatomically indistinct with no difference in MRI contrast leading to segmentation relying on heuristic geometrical rules which may lead to significant inter-study variability (Yushkevich et al., 2015a,b). Alternatively it may be that CA1 is less associated specifically with verbal episodic memory impairment but rather implicated in global cognitive impairment; to strengthen this argument, as described above we did find CA1 associated with a declining MMSE score.

In our second supplementary analysis looking at the verbal fluency cognitive domain, we identified baseline reduction in presubiculum, CA1, CA3, CA4, molecular layer, dentate gyrus, hippocampal tail and HATA. This is at odds with previous cross-sectional work (Sass et al., 1992; Carey et al., 2019) which found no association between verbal or semantic fluency and hippocampal subfields, which could be due to the longitudinal design of our study. Otherwise there have been few studies looking specifically at this cognitive domain which is known to be associated with Alzheimer’s type dementia (Henry et al., 2004).

In contrast to the cognitively declining group, the Mid-Stable group did not demonstrate the multi-subfield baseline reduction in volume compared to the High-Stable group. Previous longitudinal studies of subjects with a formal diagnosis of MCI have identified two distinct categories, those who maintain their level of cognition (MCI-Stable) and those who progress to AD (MCI-Converter). As noted, Khan et al. (2015) and Kälin et al. (2017) have reported that MCI-Stable patients have hippocampi that morphologically resemble those of healthy controls while MCI-Converter subjects have hippocampi that resemble those of patients with clinical AD. In our study the mid-stable cohort had smaller presubiculum, hippocampal tail and HATA volumes, with much less widespread atrophy than in our low-stable cohort.

### Limitations

Firstly, hippocampal subfield volumes were taken at baseline and therefore temporal direction/causality cannot be fully drawn.

Secondly, automatic segmentation of the hippocampal subfields in our study was based on T1-weighed (T1w) images rather than the preferred combination of T1 and T2 sequences. Criticisms have been recently levied against hippocampal subfield segmentation based on T1w images (Wisse et al., 2021) due to their low resolution and therefore inefficiency for automatic segmentations to accurately distinguish some specific subfields, such as the molecular layer (Iglesias et al., 2015; Wisse et al., 2021). Iglesias et al. (2015) advised use of a combination of T1w and T2w images as input for an optimal segmentation. In our MR protocol, the T1w and T2w images had considerably different in-plane resolutions (T1w were 0.9 mm isotropic; T2w were 0.58 × 0.72 × 4), which precluded combining both images to achieve a better overall segmentation. Another approach recommended by Wisse et al. (2021) is to ensure the validity of the automatic segmentation against high-reliability manual segmentation (bronze-standard approach) or direct comparison to histological samples (gold standard approach). However, validating FreeSurfer segmentation against manual segmentation is prohibitively time consuming due to its labour-intensive nature and given our relatively large dataset (*n = 482*). Manual delineation of hippocampal subfields in high-resolution images is estimated at approximately 50 h per case (Iglesias et al., 2015). While we recognize that our approach departs from optimal standards and that our findings should be interpreted with caution, our relatively large sample size, and the consistency of our methodology with respect to other large cohort studies (e.g., UK Biobank), as mentioned above, afford the opportunity for our results to be replicated in future studies.

Third we used listwise deletion. Loss to follow-up and missing data means that the study sample was overall healthier than those excluded, therefore limiting the generalizability of our findings. This does not however preclude the observed associations. The MRI sub-group of the TILDA sample is also a relatively highly functioning cohort which may limit applicability of findings to the general population.

Fourth, the number of participants who were in the Low-declining group was low. Replication of this study with larger sample size would be needed to confirm our findings.

### Strengths

To the best of our knowledge, this is the first study to assess hippocampal subfield volumes in association with changes in global and domain-specific cognitive function over a 4 year follow up, in a relatively large cohort of healthy older adults from a nationally representative population based study of ageing. Our models were also controlled for a large range of confounding covariates including head size, age, sex, education, self-reported cardiovascular diseases and events, systolic and diastolic blood pressure, smoking, alcohol intake, physical exercise, BMI, depressive symptoms, cardiovascular and antidepressant medications suggesting that these factors do not account for the observed findings.

The use of 3-Tesla MRI improves the signal-to-noise ratio of the images allowing better image resolution (Chow et al., 2015). Several other studies, including several studies which use data acquired as part of the ADNI or AddNeuroMed studies, have used 1.5 T MRI which cannot give the same anatomic resolution on T1W images (Apostolova et al., 2006; Khan et al., 2015). Chow et al. (2015), compared 1.5 T and 3 T MRI, acquired as part of the ADNI study, for mapping hippocampal atrophy, finding that 3 T images gave superior signal-to-noise ratio and detected atrophy with greater effect, although diagnostic outcomes of the two field strengths were comparable.

## Conclusion

This study reaffirms that several hippocampal studies are associated with declining cognitive function. Elucidating associations between subfield atrophy and progressive cognitive decline raises the possibility that generating predictive algorithms for identifying subjects at risk of cognitive decline is attainable.

Future research in the TILDA cohort will look to build on our findings and to generate prediction models incorporating imaging findings with other clinical predictors of cognitive decline.

## Data availability statement

The raw data supporting the conclusions of this article will be made available by the authors, without undue reservation.

## Ethics statement

The studies involving humans were approved by Trinity College Faculty of Health Sciences Research Ethics Committee, Dublin, Ireland. The studies were conducted in accordance with the local legislation and institutional requirements. The participants provided their written informed consent to participate in this study.

## Author contributions

SD: Conceptualization, Methodology, Visualization, Writing – original draft, Writing – review & editing. DC: Data curation, Methodology, Writing – review & editing. SK: Data curation, Methodology, Writing – review & editing. JM: Conceptualization, Funding acquisition, Methodology, Project administration, Supervision, Writing – review & editing. RK: Conceptualization, Funding acquisition, Methodology, Project administration, Resources, Supervision, Validation, Visualization, Writing – review & editing. CL: Conceptualization, Data curation, Formal analysis, Methodology, Project administration, Supervision, Validation, Visualization, Writing – original draft, Writing – review & editing.
