# Supplementary material for: Relationship between hippocampal subfield volumes and cognitive decline in healthy subjects
Source: Front Aging Neurosci. 2023 Dec 7;15:1284619. doi: 10.3389/fnagi.2023.1284619 (PMC10733466; doi:10.3389/fnagi.2023.1284619)
Supplement: Supplementary file 1 [file Data_Sheet_1.docx]

**SUPPLEMENTARY FIGURES AND TABLES**

**Figure S1:** Flowchart of the study sample design.

HA= home-based health assessment, MRI= magnetic resonance imaging, TIA= transient ischaemic attack, W3= wave 3, W4= wave 4, W5= wave 5

**
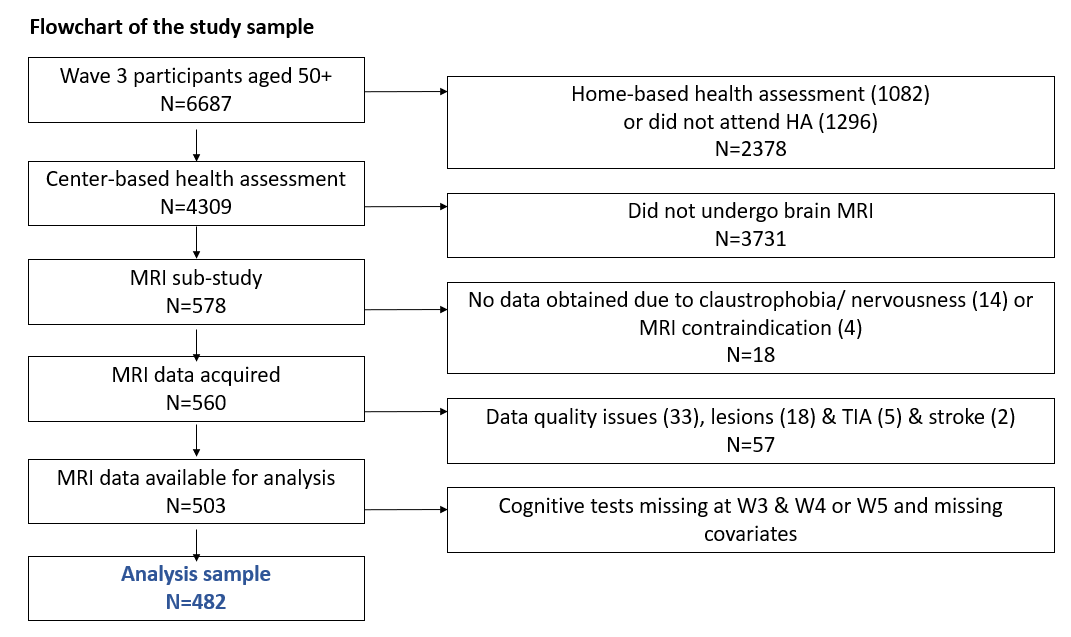
**

**Table S2:** Mini Mental State Exam (MMSE) scores at wave 3, wave 4 and wave 5 for the three cognitive trajectories: High-Stable, Mid-Stable and Low-Declining.

| **MMSE Mean (Sd)** | **High-Stable (N=315; 65%)** | **Mid-Stable (N=141; 29%)** | **Low-Declining (N=26; 5%)** |
| --- | --- | --- | --- |
| Wave 3 | 29.4 (0.6) | 27.7 (1.34) | 27.0 (2.6) |
| Wave 4 | 29.5 (0.6) | 28.0 (1.16) | 25.9 (2.2) |
| Wave 5 | 29.4 (0.8) | 28.3 (1.16) | 23.7 (2.5) |

**Figure S3:** Visual illustration of **Figure S2** Mini Mental State Exam (MMSE) scores at wave 3, wave 4 and wave 5 for the three cognitive trajectories: High-Stable, Mid-Stable and Low-Declining.

**
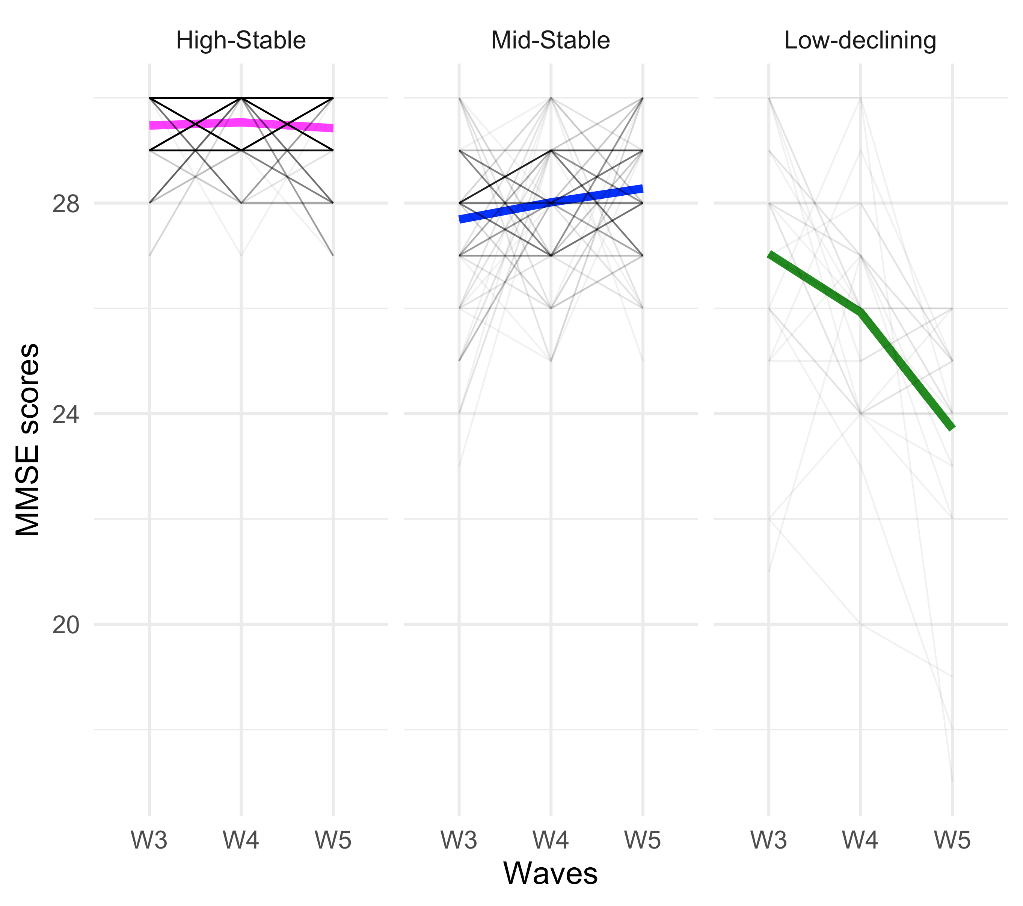
**

**Table S4:** Estimates and 95% confidence intervals of the relationship between hippocampal subfield volumes and global cognitive function trajectory group (Mini-Mental State Examination), controlled for age, sex, education and estimated Intracranial Volume (eTIV).

|  | **Estimates** | **95% Confidence Interval** | **P values** |
| --- | --- | --- | --- |
| (Intercept) | 3.36 | 2.79 – 3.93 | **<.001** |
| **Low-Declining MMSE** |  |  |  |
| CA1 | -0.34 | -0.78 – -0.02 | **.04** |
| CA3 | -0.37 | -0.75 ­­– 0.01 | .06 |
| CA4 | -0.55 | -0.93 – -0.17 | **.005** |
| Dentate Gyrus | -0.57 | -0.94 – -0.19 | **.003** |
| Fimbria | -0.11 | -0.49 – 0.27 | .57 |
| Fissure | 0.00 | -0.38 – 0.38 | .99 |
| HATA | -0.41 | -0.79 – -0.03 | **.04** |
| Molecular Layer | -0.49 | -0.87 – -0.11 | **.01** |
| Presubiculum | -0.20 | -0.81 – -0.05 | **.04** |
| Subiculum | -0.44 | -0.82 – -0.06 | **.02** |
| Hippocampal Tail | -0.53 | -0.91 – -0.15 | **.006** |
| **Mid-Stable MMSE** |  |  |  |
| CA1 | -0.007 | -0.20 – 0.18 | .94 |
| CA3 | -0.09 | -0.27 – 0.10 | .35 |
| CA4 | -0.13 | -0.32 – 0.06 | .19 |
| Dentate Gyrus | -0.15 | -0.33 – 0.04 | .13 |
| Fimbria | -0.15 | -0.34 – 0.03 | .11 |
| Fissure | 0.18 | -0.00 – 0.38 | .051 |
| HATA | -0.22 | -0.41 – -0.03 | **.02** |
| Molecular Layer | -0.17 | -0.30 – 0.07 | .22 |
| Presubiculum | -0.20 | -0.39 – -0.01 | **.04** |
| Subiculum | -0.09 | -0.28 – 0.10 | .35 |
| Hippocampal Tail | -0.20 | -0.39 – -0.01 | **.04** |
| **Covariates** |  |  |  |
| Age | -0.04 | -0.04 – -0.03 | **<.001** |
| Sex | -0.27 | -0.41 – -0.13 | **<.001** |
| Education | -0.05 | -0.02 – -0.03 | 0.25 |
| eTIV | 0.28 | 0.20 – 0.35 | **<.001** |

P values <.05 highlighted in bold. CA1 = cornu ammonis 1, CA3= cornu ammonis 3, CA4= cornu ammonis 4, HATA= hippocampus amygdala transition area, eTIV= estimated total intracranial volume.

**Table S5:** Estimates and 95% confidence intervals of the relationship between hippocampal subfield volumes and global cognitive function trajectory group (Mini-Mental State Examination), controlled for age, sex, education, estimated Intracranial Volume (eTIV) and extended lifestyle covariates.

| **Covariates** | **Estimates** | **95% Confidence Interval** | **P values** |
| --- | --- | --- | --- |
| (intercept) | 3.53 | 2.67 – 4.39 | **<.001** |
| **Low-Declining MMSE** |  |  |  |
| CA1 | -0.40 | -0.78 – -0.02 | **.04** |
| CA3 | -0.37 | -0.75 – 0.01 | .06 |
| CA4 | -0.55 | -0.93 – -0.17 | **.005** |
| Dentate Gyrus | -0.57 | -0.95 – -0.19 | **.004** |
| Fimbria | -0.11 | -0.49 – 0.27 | .57 |
| Fissure | 0.00 | -0.38 – 0.38 | .99 |
| HATA | -0.41 | -0.79 – -0.03 | **.04** |
| Molecular Layer | -0.50 | -0.87 – -0.11 | **.01** |
| Presubiculum | -0.43 | -0.81 – -0.05 | **.03** |
| Subiculum | -0.44 | -0.82 – -0.06 | **.02** |
| Hippocampal Tail | -0.53 | -0.91 – -0.15 | **.006** |
| **Mid-Stable MMSE** |  |  |  |
| CA1 | -0.01 | -0.20 – 0.18 | .94 |
| CA3 | -0.09 | -0.28 – 0.10 | .35 |
| CA4 | -0.13 | -0.32 – 0.06 | .19 |
| Dentate Gyrus | -0.15 | -0.33 – 0.04 | .13 |
| Fimbria | -0.15 | -0.34 – 0.03 | .11 |
| Fissure | 0.19 | -0.00 – 0.38 | .051 |
| HATA | -0.22 | -0.41 – -0.03 | **.02** |
| Molecular Layer | -0.11 | -0.31 – 0.07 | .22 |
| Presubiculum | 0.20 | -0.39 – -0.01 | **.04** |
| Subiculum | -0.09 | -0.28 – 0.10 | .35 |
| Hippocampal Tail | -0.20 | -0.39 – -0.01 | **.04** |
| **Covariates** |  |  |  |
| Age | -0.05 | -0.06 – -0.04 | **<.001** |
| Sex | -0.26 | -0.40 – -0.11 | **<.001** |
| Education | -0.04 | -0.12 – 0.03 | .29 |
| eTIV | 0.28 | 0.21 – 0.35 | **<.001** |
| Systolic BP | -0.01 | -0.02 – -0.00 | **.009** |
| Diastolic BP | -0.01 | -0.02 – -0.00 | **.04** |
| Physical Exercise Mid | 0.00 | -0.13 – 0.13 | .99 |
| Physical Exercise High | -0.09 | -0.24 – 0.05 | .22 |
| Alcohol | -0.10 | -0.29 – 0.08 | .30 |
| Smoking Current | -0.03 | -0.15 – 0.08 | .60 |
| Smoking Past | -0.17 | -0.41 – 0.07 | .18 |
| BMI | 0.00 | -0.01 – 0.02 | .57 |
| Depressive Symptoms | 0.01 | -0.01 – 0.02 | .40 |
| CVD Conditions | 0.01 | -0.12 – 0.14 | .89 |
| Antidepressant use | -0.15 | -0.37 – 0.08 | .21 |
| Antihypertensive use | -0.04 | -0.17 – 0.10 | .61 |

P values <.05 highlighted in bold. CA1 = cornu ammonis 1, CA3= cornu ammonis 3, CA4= cornu ammonis 4, HATA= hippocampus amygdala transition area, eTIV= estimated total intracranial volume, BP= blood pressure, BMI= body mass index, CVD= cardiovascular disease.

**Table S6:** Estimates and 95% confidence intervals of the relationship between hippocampal subfield volumes and verbal memory (delayed recall), controlled for age, sex, education and estimated Intracranial Volume (eTIV).

| **Covariates** | **Estimates** | **95% Confidence Interval** | **P values** |
| --- | --- | --- | --- |
| (intercept) | 3.33 | 2.74 – 3.92 | **<.001** |
| **Low-Declining MMSE** |  |  |  |
| CA1 | -0.14 | -0.37 – 0.10 | .26 |
| CA3 | -0.25 | -0.49 – -0.13 | **.04** |
| CA4 | -0.32 | -0.56 – -0.09 | **.008** |
| Dentate Gyrus | -0.35 | -0.59 – -0.11 | **.004** |
| Fimbria | -0.50 | -0.73 – -0.26 | **<.001** |
| Fissure | 0.17 | -0.07 – 0.41 | .16 |
| HATA | -0.39 | -0.63 – -0.15 | **.001** |
| Molecular Layer | -0.32 | -0.56 – -0.08 | **.008** |
| Presubiculum | -0.37 | -0.60 – -0.13 | **.003** |
| Subiculum | -0.27 | -0.51 – -0.04 | **.02** |
| Hippocampal Tail | -0.36 | -0.60 – -0.12 | **.003** |
| **Mid-Stable MMSE** |  |  |  |
| CA1 | 0.04 | -0.15 – 0.23 | .58 |
| CA3 | 0.01 | -0.18 – 0.20 | .89 |
| CA4 | -0.05 | -0.24 – 0.14 | .57 |
| Dentate Gyrus | -0.06 | -0.25 – 0.13 | .55 |
| Fimbria | -0.09 | -0.28 – 0.10 | .37 |
| Fissure | 0.31 | 0.12 – 0.50 | **.001** |
| HATA | -0.06 | -0.25 – 0.13 | .54 |
| Molecular Layer | -0.04 | -0.23 – 0.15 | .66 |
| Presubiculum | -0.13 | -0.32 – 0.06 | .17 |
| Subiculum | -0.03 | -0.22 – 0.16 | .73 |
| Hippocampal Tail | -0.08 | -0.27 – 0.11 | .41 |
| **Covariates** |  |  |  |
| Age | -0.04 | -0.05 – -0.03 | **<.001** |
| Sex | -0.26 | -0.40 – -0.12 | **<.001** |
| Education | -0.03 | -0.11 – 0.04 | .42 |
| eTIV | 0.29 | 0.22 – 0.36 | **<.001** |

P values <.05 highlighted in bold. CA1 = cornu ammonis 1, CA3= cornu ammonis 3, CA4= cornu ammonis 4, HATA= hippocampus amygdala transition area, eTIV= estimated total intracranial volume.

**Table S7:** Estimates and 95% confidence intervals of the relationship between hippocampal subfield volumes and verbal memory (delayed recall), controlled for age, sex, education, estimated Intracranial Volume (eTIV) and extended lifestyle covariates.

| **Covariates** | **Estimates** | **95% Confidence Interval** | **P values** |
| --- | --- | --- | --- |
| (intercept) | 3.52 | 2.65 – 4.39 | **<.001** |
| **Low-Declining MMSE** |  |  |  |
| CA1 | -0.14 | -0.37 – 0.10 | .26 |
| CA3 | -0.25 | -0.49 – -0.01 | **.04** |
| CA4 | -0.32 | -0.56 – -0.09 | **.008** |
| Dentate Gyrus | -0.35 | -0.59 – -0.11 | **.004** |
| Fimbria | -0.50 | -0.73 – -0.26 | **<.001** |
| Fissure | 0.17 | -0.07 –0.41 | .16 |
| HATA | -0.39 | -0.63 – -0.15 | **.001** |
| Molecular Layer | -0.32 | -0.56 – -0.08 | **.008** |
| Presubiculum | -0.37 | -0.60 – -0.13 | **.003** |
| Subiculum | -0.27 | -0.51 – -0.04 | **.02** |
| Hippocampal Tail | -0.36 | -0.60 – -0.12 | **.003** |
| **Mid-Stable MMSE** |  |  |  |
| CA1 | -0.04 | -0.15 – 0.23 | .68 |
| CA3 | 0.01 | -0.18 – 0.20 | .89 |
| CA4 | -0.05 | -0.24 – 0.14 | .57 |
| Dentate Gyrus | -0.06 | -0.25 – 0.14 | .55 |
| Fimbria | -0.09 | -0.28 – 0.10 | **.37** |
| Fissure | 0.31 | 0.12 – 0.50 | **.001** |
| HATA | -0.06 | -0.25 – 0.13 | .54 |
| Molecular Layer | -0.04 | -0.23 – 0.15 | .66 |
| Presubiculum | -0.13 | -0.32 – 0.06 | .17 |
| Subiculum | -0.03 | -0.22 – 0.16 | .73 |
| Hippocampal Tail | -0.08 | -0.27 – 0.11 | .41 |
| **Covariates** |  |  |  |
| Age | -0.05 | -0.06 – -0.04 | **<.001** |
| Sex | -0.24 | -0.39 – -0.10 | **.001** |
| Education | -0.03 | -0.10 – 0.05 | .51 |
| eTIV | 0.29 | 0.22 – 0.36 | **<.001** |
| Systolic BP | -0.01 | 0.00 – 0.01 | **.01** |
| Diastolic BP | -0.01 | -0.02 – -0.00 | **.05** |
| Physical Exercise Mid | 0.01 | -0.12 – 0.14 | .91 |
| Physical Exercise High | -0.08 | -0.23 – 0.07 | .28 |
| Alcohol | -0.11 | -0.30 – 0.08 | .28 |
| Smoking Current | -0.03 | -0.14 – 0.09 | .67 |
| Smoking Past | -0.14 | -0.38 – 0.10 | .27 |
| BMI | 0.00 | -0.01 – 0.02 | .64 |
| Depressive Symptoms | 0.00 | -0.01 – 0.02 | .58 |
| CVD Conditions | 0.01 | -0.13 – 0.14 | .92 |
| Antidepressant use | -0.15 | -0.37 – 0.08 | .21 |
| Antihypertensive use | -0.02 | -0.16 – 0.12 | .78 |

P values <.05 highlighted in bold. CA1 = cornu ammonis 1, CA3= cornu ammonis 3, CA4= cornu ammonis 4, HATA= hippocampus amygdala transition area, eTIV= estimated total intracranial volume, BP= blood pressure, BMI= body mass index, CVD= cardiovascular disease.

**Table S8:** Estimates and 95% confidence intervals of the relationship between hippocampal subfield volumes and verbal fluency, controlled for age, sex, education and estimated Intracranial Volume (eTIV).

| **Covariates** | **Estimates** | **95% Confidence Interval** | **P values** |
| --- | --- | --- | --- |
| (intercept) | 3.31 | 2.71 – 3.91 | **<.001** |
| **Low-Declining MMSE** |  |  |  |
| CA1 | -0.23 | -0.46 – -0.00 | **.05** |
| CA3 | -0.27 | -0.49 – -0.04 | **.02** |
| CA4 | -0.31 | -0.54 – -0.09 | **.007** |
| Dentate Gyrus | -0.32 | -0.54 – -0.09 | **.006** |
| Fimbria | -0.15 | -0.38 – 0.08 | .19 |
| Fissure | 0.06 | -0.16 – 0.29 | .59 |
| HATA | -0.30 | -0.53 – -0.07 | **.01** |
| Molecular Layer | -0.27 | -0.50 – -0.04 | **.02** |
| Presubiculum | -0.23 | -0.50 – -0.05 | **.02** |
| Subiculum | -0.17 | -0.40 – 0.06 | .14 |
| Hippocampal Tail | -0.27 | -0.50 – -0.04 | **.02** |
| **Mid-Stable MMSE** |  |  |  |
| CA1 | 0.20 | -0.42 – 0.01 | .07 |
| CA3 | 0.23 | -0.44 – -0.01 | **.04** |
| CA4 | -0.19 | -0.40 – 0.03 | .10 |
| Dentate Gyrus | -0.19 | -0.40 – 0.03 | .09 |
| Fimbria | -0.05 | -0.27 – 0.17 | .64 |
| Fissure | -0.18 | -0.39 – 0.04 | .12 |
| HATA | -0.08 | -0.29 – 0.14 | .49 |
| Molecular Layer | -0.15 | -0.36 – 0.07 | .19 |
| Presubiculum | -0.07 | -0.28 – 0.15 | .55 |
| Subiculum | -0.11 | -0.32 – 0.11 | .34 |
| Hippocampal Tail | -0.13 | -0.35 – 0.09 | .25 |
| **Covariates** |  |  |  |
| Age | -0.04 | -0.05 – -0.04 | **<.001** |
| Sex | -0.25 | -0.39 – -0.11 | **<.001** |
| Education | -0.03 | -0.11 – 0.04 | .39 |
| eTIV | 0.29 | 0.22 – 0.36 | **<.001** |

P values <.05 highlighted in bold. CA1 = cornu ammonis 1, CA3= cornu ammonis 3, CA4= cornu ammonis 4, HATA= hippocampus amygdala transition area, eTIV= estimated total intracranial volume.

**Table S9:** Estimates and 95% confidence intervals of the relationship between hippocampal subfield volumes and verbal fluency, controlled for age, sex, education, estimated Intracranial Volume (eTIV) and extended lifestyle covariates.

| **Covariates** | **Estimates** | **95% Confidence Interval** | **P values** |
| --- | --- | --- | --- |
| (intercept) | 3.31 | 2.71 – 3.91 | **<.001** |
| **Low-Declining MMSE** |  |  |  |
| CA1 | -0.23 | -0.46 – -0.00 | **.05** |
| CA3 | -0.27 | -0.49 – -0.04 | **.02** |
| CA4 | -0.31 | -0.54 – -0.09 | **.007** |
| Dentate Gyrus | -0.32 | -0.54 – -0.09 | **.006** |
| Fimbria | -0.15 | -0.38 – 0.08 | .19 |
| Fissure | 0.06 | -0.16 – 0.29 | .59 |
| HATA | -0.30 | -0.53 – -0.07 | **.01** |
| Molecular Layer | -0.27 | -0.50 – -0.04 | **.02** |
| Presubiculum | -0.27 | -0.50 – -0.05 | **.02** |
| Subiculum | -0.17 | -0.40 – 0.06 | .14 |
| Hippocampal Tail | -0.27 | -0.50 – -0.04 | **.02** |
| **Mid-Stable MMSE** |  |  |  |
| CA1 | -0.20 | -0.42 – 0.01 | .07 |
| CA3 | -0.23 | -0.44 – - 0.01 | **.04** |
| CA4 | -0.19 | -0.40 – 0.03 | .10 |
| Dentate Gyrus | -0.19 | -0.40 – 0.03 | .09 |
| Fimbria | -0.05 | -0.27 –0.17 | .64 |
| Fissure | -0.17 | -0.39 – 0.04 | .12 |
| HATA | -0.08 | -0.29 – 0.14 | .49 |
| Molecular Layer | -0.15 | -0.36 – 0.07 | .19 |
| Presubiculum | -0.07 | -0.28 – 0.15 | .55 |
| Subiculum | -0.11 | -0.32 – 0.11 | .34 |
| Hippocampal Tail | -0.13 | -0.35 – 0.09 | .25 |
| **Covariates** |  |  |  |
| Age | -0.05 | -0.06 – -0.04 | **<.001** |
| Sex | -0.23 | -0.38 – -0.09 | **.002** |
| Education | -0.03 | -0.11 – 0.05 | .44 |
| eTIV | 0.29 | 0.22 – 0.36 | **<.001** |
| Systolic BP | 0.01 | 0.00 – 0.01 | **.01** |
| Diastolic BP | -0.01 | -0.02 – -0.00 | **.04** |
| Physical Exercise Mid | 0.01 | -0.12 – 0.14 | .89 |
| Physical Exercise High | -0.09 | -0.23 – 0.06 | .27 |
| Alcohol | -0.11 | -0.30 – 0.08 | .27 |
| Smoking Current | -0.03 | -0.14 – 0.08 | .59 |
| Smoking Past | -0.13 | -0.37 – 0.11 | .28 |
| BMI | 0.00 | -0.01 – 0.02 | .60 |
| Depressive Symptoms | 0.00 | -0.01 – 0.02 | .59 |
| CVD Conditions | 0.00 | -0.13 – 0.13 | .99 |
| Antidepressant use | -0.17 | -0.39 – 0.06 | .15 |
| Antihypertensive use | -0.02 | -0.16 – 0.11 | .75 |

P values <.05 highlighted in bold. CA1 = cornu ammonis 1, CA3= cornu ammonis 3, CA4= cornu ammonis 4, HATA= hippocampus amygdala transition area, eTIV= estimated total intracranial volume, BP= blood pressure, BMI= body mass index, CVD= cardiovascular disease.
